# Antibiotic Review Kit for Hospitals (ARK-Hospital): study protocol for a stepped-wedge cluster-randomised controlled trial

**DOI:** 10.1186/s13063-019-3497-y

**Published:** 2019-07-11

**Authors:** Ann Sarah Walker, Eric Budgell, Magda Laskawiec-Szkonter, Katy Sivyer, Sarah Wordsworth, Jack Quaddy, Marta Santillo, Adele Krusche, Laurence S. J. Roope, Nicole Bright, Fiona Mowbray, Nicola Jones, Kieran Hand, Najib Rahman, Melissa Dobson, Emma Hedley, Derrick Crook, Mike Sharland, Chris Roseveare, F. D. Richard Hobbs, Chris Butler, Louella Vaughan, Susan Hopkins, Lucy Yardley, Timothy E. A. Peto, Martin J. Llewelyn, on behalf of the ARK trial team

**Affiliations:** 1 Nuffield Department of Medicine, University of Oxford, Oxford, UK; 2 Oxford Respiratory Trials Unit, University of Oxford, Oxford, UK; 3 Centre for Clinical and Community Applications of Health Psychology, University of Southampton, Southampton, UK; 4 Health Economics Research Centre, Nuffield Department of Population Health, University of Oxford, Oxford, UK; 5 Oxford University Hospitals NHS Foundation Trust, Oxford, UK; 6 University of Southampton, Southampton, UK; 7 University Hospital Southampton NHS Trust, Southampton, UK; 8 St George’s, University of London, London, UK; 9 Southern Health NHS Foundation Trust, Southampton, UK; 10 Department of Primary Care Health Sciences, University of Oxford, Oxford, UK; 11 The Nuffield Trust, London, UK; 12 Royal Free London NHS Foundation Trust, London, UK; 13 School of Psychological Science, University of Bristol, Clifton, UK; 14 National Infection Service, Public Health England, London, UK; 15 Brighton and Sussex Medical School, Brighton, UK

**Keywords:** Antibiotic prescribing, Hospitals, Antimicrobial stewardship

## Abstract

**Background:**

To ensure patients continue to get early access to antibiotics at admission, while also safely reducing antibiotic use in hospitals, one needs to target the continued need for antibiotics as more diagnostic information becomes available. UK Department of Health guidance promotes an initiative called ‘Start Smart then Focus’: early effective antibiotics followed by active ‘review and revision’ 24–72 h later. However in 2017, < 10% of antibiotic prescriptions were discontinued at review, despite studies suggesting that 20–30% of prescriptions could be stopped safely.

**Methods/design:**

Antibiotic Review Kit for Hospitals (ARK-Hospital) is a complex ‘review and revise’ behavioural intervention targeting healthcare professionals involved in antibiotic prescribing or administration in inpatients admitted to acute/general medicine (the largest consumers of non-prophylactic antibiotics in hospitals). The primary study objective is to evaluate whether ARK-Hospital can safely reduce the total antibiotic burden in acute/general medical inpatients by at least 15%. The primary hypotheses are therefore that the introduction of the behavioural intervention will be non-inferior in terms of 30-day mortality post-admission (relative margin 5%) for an acute/general medical inpatient, and superior in terms of defined daily doses of antibiotics per acute/general medical admission (co-primary outcomes). The unit of observation is a hospital organisation, a single hospital or group of hospitals organised with one executive board and governance framework (National Health Service trusts in England; health boards in Northern Ireland, Wales and Scotland). The study comprises a feasibility study in one organisation (phase I), an internal pilot trial in three organisations (phase II) and a cluster (organisation)-randomised stepped-wedge trial (phase III) targeting a minimum of 36 organisations in total. Randomisation will occur over 18 months from November 2017 with a further 12 months follow-up to assess sustainability. The behavioural intervention will be delivered to healthcare professionals involved in antibiotic prescribing or administration in adult inpatients admitted to acute/general medicine. Outcomes will be assessed in adult inpatients admitted to acute/general medicine, collected through routine electronic health records in all patients.

**Discussion:**

ARK-Hospital aims to provide a feasible, sustainable and generalisable mechanism for increasing antibiotic stopping in patients who no longer need to receive them at ‘review and revise’.

**Trial registration:**

ISRCTN Current Controlled Trials, ISRCTN12674243. Registered on 10 April 2017.

**Electronic supplementary material:**

The online version of this article (10.1186/s13063-019-3497-y) contains supplementary material, which is available to authorized users.

## Background

Antimicrobial resistance is one of the most important global public health risks recognised by the UK government [1] and the World Health Organization (WHO) [2], with increasing impacts on health [3]. It is increasing year-on-year, particularly in common Gram-negative bacteria [4, 5]; for example, the incidence of *Escherichia coli* bloodstream infections resistant to the most commonly used antibiotics has quadrupled over the last decade in Oxfordshire [6].

The only way to reduce the selective pressure on bacteria to develop resistance is to reduce antibiotic use [7, 8]. Resistant strains emerge rapidly in faecal [9] and nasal flora [10–12] during antibiotic treatment. Detectable resistance in these carried organisms wanes over time, but it can persist [13] and is still associated with developing antibiotic-resistant infections [14, 15], as is prior antibiotic use [15, 16]. Reducing population-level antibiotic consumption can significantly reduce drug resistance [17], although this is not a simple cause-effect relationship. Most bacterial resistance is due to genes carried on mobile genetic elements which move within and between bacterial species. Thus, removing selection pressure from one antibiotic will not always lead to loss of resistance, e.g. if other beneficial genes (or genes conferring resistance to other antibiotics still in use) are co-carried on the same mobile element [18–20]. Nevertheless, countries with higher antibiotic consumption generally have higher rates of antibiotic-resistant infections [21].

Whilst hospital prescribing accounts for < 20% of the antibiotics consumed in England, around two-thirds of prescriptions for ‘broad-spectrum’ agents (active against a wide variety of bacteria, including some antibiotic-resistant strains) are made in hospitals [5]. These broad-spectrum antibiotics have the greatest potential to drive future resistance, and, in contrast to primary care, hospital prescribing continues to increase (by 7% over 2013–2018 [5]). Aside from resistance, antibiotics are also associated with non-trivial rates (~ 20%) of adverse drug reactions [22].

Avoiding or delaying prescription are two strategies that successfully lower antibiotic consumption in primary care [23, 24]. However, in contrast to primary care patients, a considerable proportion of those presenting to hospital will genuinely have bacterial infections and need antibiotics. If a patient has a life-threatening bacterial infection, delays in administering effective antibiotics of even one hour increase mortality risk [25]. The challenge is accurately identifying these patients and their infecting microorganisms, which is difficult and takes time. This provides the rationale for prescribing broad-spectrum antibiotics whenever infection is suspected at admission (see e.g. ‘Surviving Sepsis’ [26]), since these should kill any infecting bacteria whilst (in theory) not harming patients without bacterial infections.

To ensure patients continue to get early access to antibiotics at admission, the safe reduction of antibiotic use in hospitals therefore needs to target the continued need for, and choice of, antibiotics as more diagnostic information becomes available. 2011 Department of Health (DH) guidance promotes an initiative called ‘Start Smart then Focus’ [27, 28]: early effective antibiotics followed by active ‘review and revision’ 24–72 h later. Five options are recommended: stop; switch from intravenous to oral administration; continue and review again; change (if possible to antibiotics with narrower spectrum of activity); or move to outpatient intravenous antibiotic therapy.

However, a 2013 survey of National Health Service (NHS) antibiotic pharmacists found that although 95% of 105 responding English trusts included early antibiotic review in their policies/guidelines, only 48% conducted any compliance monitoring [29]. Where compliance was monitored, relatively few (< 20%) prescription changes of any kind were made. This agrees with studies reporting clinicians’ reluctance to modify antibiotic prescribing decisions made by others, even when a patient’s clinical status has evolved [30] and reflects the complexity of decision making on antibiotic prescribing [31]. Trust policies frequently recommended antibiotic durations of ≥ 7 days [29], suggesting lack of review could lead to substantial overuse. Even by 2017, < 10% of antibiotic prescriptions were discontinued at review [32], despite studies suggesting that 20–30% of prescriptions could be stopped safely [33]. Despite considerable variation in local guideline-recommended antibiotic duration and practice [29], there are no data showing whether or not trusts which routinely use fewer antibiotics have higher rates of adverse outcomes.

This failure of prescribers to change or stop antibiotics is perhaps unsurprising given the lack of evidence to inform how ‘review and revise’ should be implemented. Of 221 hospital antimicrobial stewardship interventions included in a Cochrane review [34], only 14 even collected duration of antibiotic therapy as an analysable outcome, let alone directly targeted it; many of these studies evaluated the introduction of procalcitonin testing as a mechanism to reduce antibiotic durations. Other interventions included introducing guidelines/policies and education/feedback. Interventions specifically designed to reduce antibiotic duration were not evaluated as a category, but overall duration of antibiotic treatment decreased by 1.95 days with stewardship interventions. One large study (*n* = 462) [35] found an intervention incorporating face-to-face pharmacist feedback significantly reduced antibiotic durations for lower respiratory tract infections in intensive care. However, another study found no impact of prescription review by telephone [36], illustrating the importance of specific intervention components.

The challenge is developing feasible, sustainable and generalisable ‘review and revise’ interventions given the complexity of hospital antibiotic prescribing. Doctors need to balance the risks of under-treating infection in patients against risks of antibiotic-resistant infections in the future, making practical choices about specific drug, dose and duration given clinical indication, within the cultural context of multidisciplinary secondary care [31]. In the near future, faster and more accurate diagnostic tests for infection will likely become available, reducing the time to identify an infecting organism and its antibiotic susceptibilities. It is highly unlikely that these tests will remove the need to prescribe broad-spectrum antibiotics for severely ill patients at admission, but they will bring the time frame for ‘review and revise’ decisions forwards and increase potential gains from optimally implementing this ‘review and revise’ approach.

## Methods

### Objective

The primary objective of ARK-Hospital is to evaluate whether a multifaceted ‘review and revise’ behavioural intervention targeting healthcare professionals involved in antibiotic prescribing or administration in inpatients admitted to acute/general medicine (the largest consumers of non-prophylactic antibiotics in hospitals [37]) can safely reduce total antibiotic burden in acute/general medical inpatients by at least 15%. The primary hypotheses are therefore that the introduction of the behavioural intervention will be non-inferior in terms of 30-day mortality post-admission for an acute/general medical inpatient and superior in terms of defined daily doses (DDDs) of antibiotics per acute/general medical admission.

### Design

The study comprises three phases: a feasibility study (phase I), an internal pilot trial (phase II) and a cluster (organisation)-randomised stepped-wedge trial (phase III) in which each organisation acts as its own control.

In the phase I feasibility study, the intervention was implemented in one organisation on 18 April 2017 to test the feasibility and acceptability of trial procedures (e.g. clinical research governance, trial documents), the trial intervention (e.g. usage, engagement, adherence, completion, fidelity) and data collection procedures (e.g. audit results, questionnaires, electronic health record data). (Cross ELA, et al.: Adaptation and Implementation of the ARK (Antibiotic Review Kit) Intervention to Safely and Substantially Reduce Antibiotic Use in Hospitals: a Feasibility Study, submitted. [38])

The phase II internal pilot formed the first phase of the substantive trial evaluating the impact of the intervention package on healthcare professionals and inpatients/carers. The intervention was iteratively implemented in three organisations between 25 September 2017 and 20 November 2017, using the ACCEPT approach [39] to identify necessary refinements and as the basis for the decision to include long-term follow-up from these phase II pilot sites within the main trial evaluation (phase III).

In the phase III main trial, the intervention is being implemented in two to four organisations per month over 18 months from 12 February 2018 to 24 June 2019, to reach the total sample size of a minimum of 36 organisations for evaluation (including phase II internal pilot sites). A further 12 months’ follow-up in all organisations will assess the sustainability of any intervention effect.

The unit of randomisation is the organisation, a single or group of hospital(s) organised with one executive board and governance framework. In England these are called *trusts*; in Northern Ireland, Scotland and Wales they are called *health boards*. Organisation is the unit of randomisation because the intervention targets healthcare professionals who frequently move between teams within an organisation. Patient- or ward/team-level randomisation is therefore infeasible because of a high certainty of contamination. A stepped-wedge design is more efficient for the same number of units than a parallel group cluster-randomised trial when the intra-class correlation coefficient is relatively large and the cluster sizes are large, because each unit acts as its own control, reducing variability [40]. Organisations are very large units of randomisation, but as explained previously, this is necessary because clinical teams are shared across organisations so there is no smaller unit of randomisation that will avoid contamination. Because antibiotic prescribing activities are based on local organisation guidelines and culture, we also expect within-organisation variation to be much smaller than between-organisation variation. Thus, we anticipate that the stepped-wedge design will be more efficient for this specific evaluation study. The stepped-wedge design also ensures that all participating organisations have the opportunity to receive the intervention at some point. This is important given the strong interest from organisations in interventions which could reduce antibiotic consumption, in the face of the emphasis on reducing inappropriate antibiotic use in hospitals from DH and wider government and financial incentives supporting such reductions [41, 42].

**Fig. 1:**
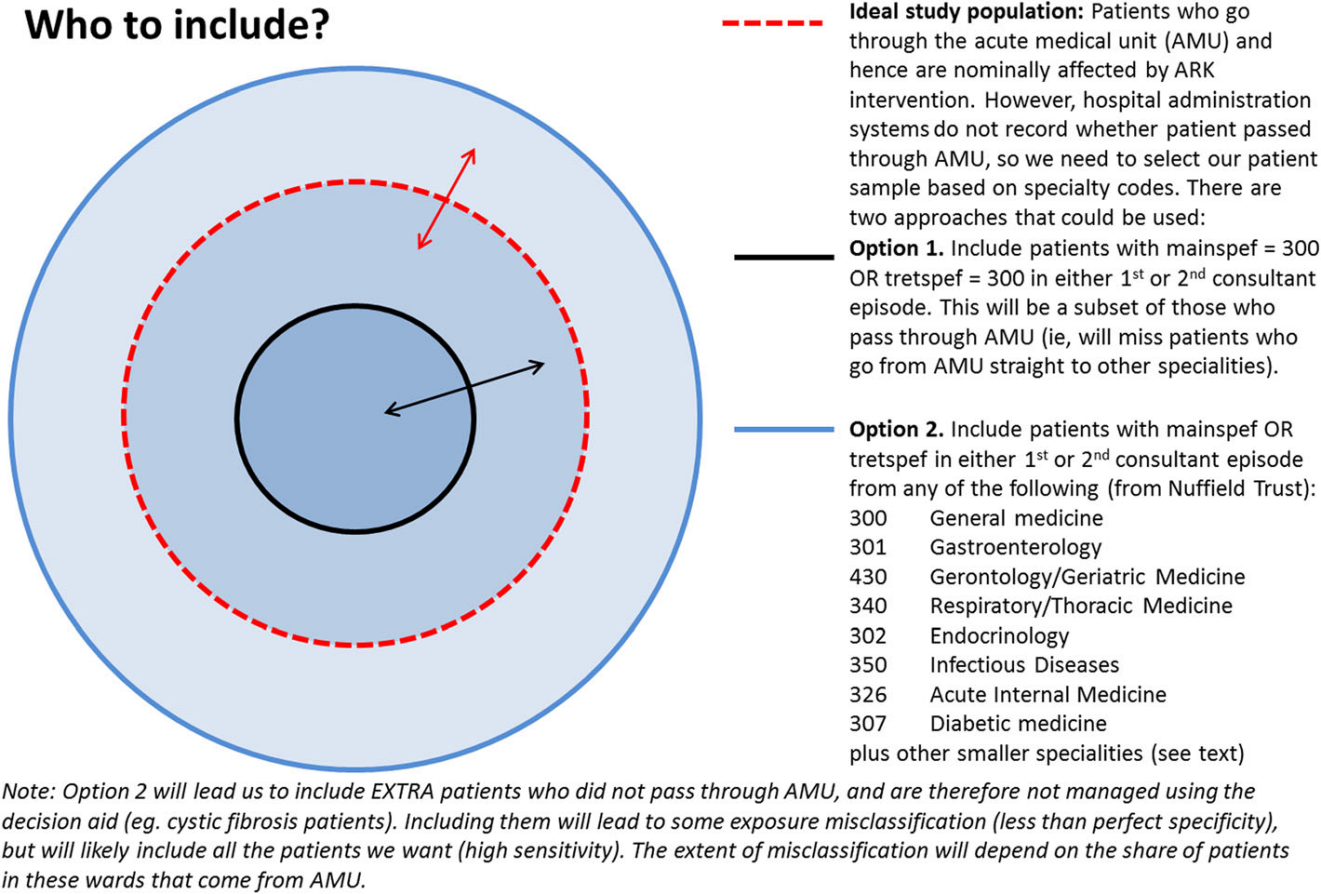
Defining the patient population from routinely collected electronic health records

**Fig. 2:**
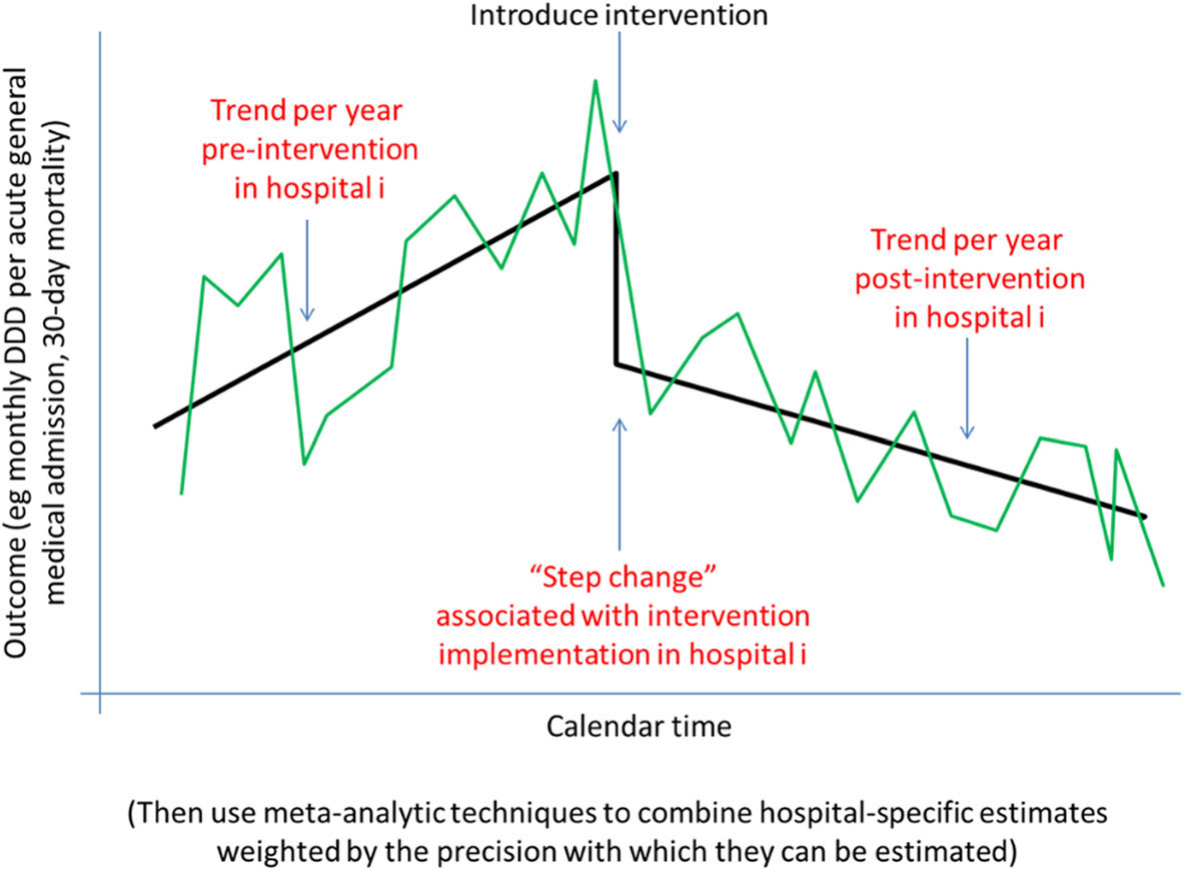
Analysis model

**Fig. 3:**
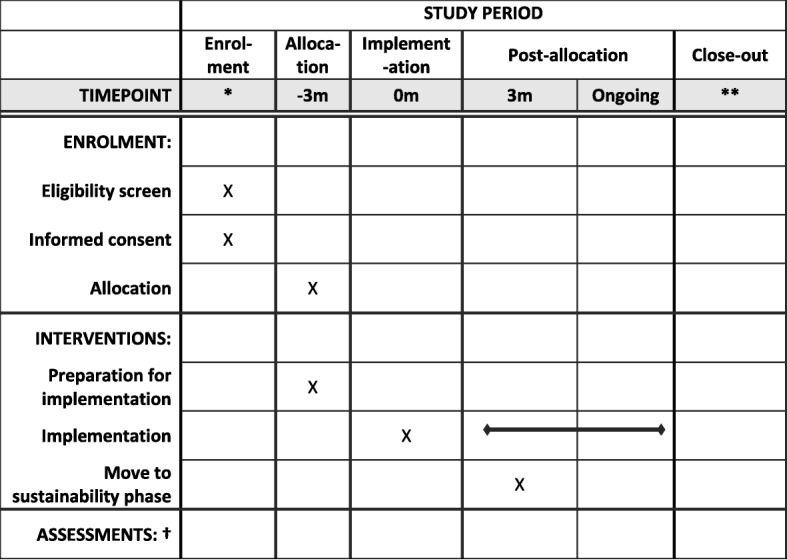
ARK schedule of enrolment, interventions, and assessments (SPIRIT figure). * Healthcare organisations are recruited to the study before enrolment but at varying calendar times, based on the fact that they can provide historical patient electronic healthcare record data pre-implementation. They are allocated an implementation date based on the stepped-wedge design. ** Follow-up finishes 12 months after the last randomised organisation implements the intervention, estimated June 2020. † There are no formal assessments. Outcomes are assessed using routinely collected electronic health records from patients admitted to acute/general medicine over the entire study period, pooled periodically during the trial

**Fig. 4:**
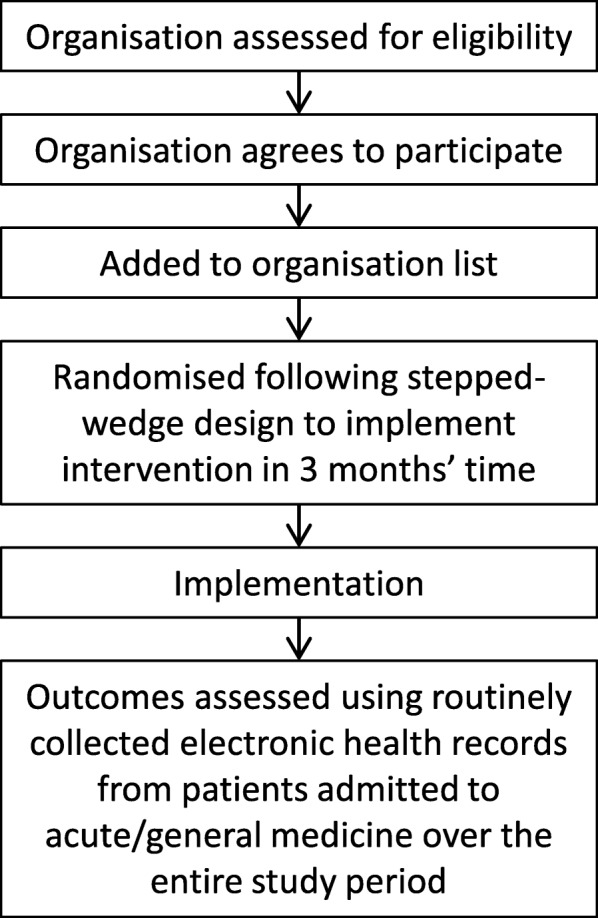
SPIRIT 2013 Checklist: Recommended items to address in a clinical trial protocol and related documents

Figure 1 shows how the patient population is defined from health records. Figure 2 illustrates the analysis model. Figure 3 shows the ARK schedule of enrolment, interventions and assessments and Fig. 4 the flow diagram. The Standard Protocol Items: Recommendations for Interventional Trials (SPIRIT) checklist is provided as Additional file 1.

### Inclusion/exclusion criteria

Organisations from England and the three devolved nations (Northern Ireland, Wales and Scotland) are eligible if they are (1) organisations admitting patients to (adult) acute/general medicine (the target patient population) (acute or district general trust in England); (2) willing to implement the intervention in healthcare professionals involved in antibiotic prescribing or administration in adult acute/general medicine and able to identify a local ‘champion’ to lead this; and (3) able to provide the required routine electronic data on adult patients admitted to acute/general medicine over the required time periods (see the discussion in subsequent sections). There are no exclusion criteria. Organisations are identified and approached through professional networks, the Society for Acute Medicine, through the clinical research networks and through word of mouth. Study sites are listed on http://www.arkstudy.ox.ac.uk/about/.

### Intervention

This protocol will test a complex behavioural intervention for healthcare professionals designed using a ‘person-based approach’ [43], based on stakeholder workshops and qualitative studies with healthcare professionals and patients, to increase the acceptability, feasibility and effectiveness of the ‘review and revise’ component of the DH ‘Start Smart then Focus’ strategy. The intervention includes several components (Santillo M, et al.: Intervention Planning for the ARK (Antibiotic Review Kit) Intervention: a Digital and Behavioural Intervention to Safely Review and Reduce Antibiotic Prescriptions in Acute and General Medicine, submitted [44]). They are:

- An evidence-based educational and motivational website (with mobile-friendly version) including training in the ARK decision aid (‘online training’)
- The ARK decision aid to classify initial antibiotic prescriptions based on risk of infection
- Data collection on rates of review and revise, use of the decision aid and rates of stopping, alongside regular and supportive discussions with clinical teams (e.g. through peer-led seminars or ward rounds) to support staff with implementation and address any barriers or concerns
- A patient leaflet which healthcare professionals can provide to acute/general medical inpatients and their carers to inform and reassure patients that their antibiotics may be stopped, without requiring increased consultation time, and to instruct them to ensure healthcare professionals are aware if their symptoms worsen. (Mowbray F, et al.: Patient Engagement with Antibiotic Messaging in Secondary Care: a Qualitative Study of the ‘Review & Revise’ Experience, submitted [45]).

The intervention includes an implementation toolkit to help the local champion and the implementation team adopt and adapt the intervention to their local structures. Relevant local factors include how acute/general medical services are provided (e.g. acute medical unit, clinical decision unit, emergency department); when/if staff change post the initial admission ‘take’ round; who is involved in decisions about continuing or not continuing antibiotics (emergency department, specialists, acute/general physicians); and local prescribing systems (e.g. paper vs electronic).

To avoid contamination, complete information about the intervention is released to the local champion at the point of randomisation, which is 3 months before anticipated organisation-wide implementation. The intervention is not blinded; patients who receive the leaflet or who are aware of study posters will also not be blinded.

### Randomisation

The phase III main trial uses an organisation-randomised stepped-wedge design in which interventions are successively implemented across different organisations over time.

The stepped-wedge cluster-randomised main trial uses calendar-time blocked randomisation. The randomisation list was drawn up in blocks of 6 before the trial started. Organisations were added to a separate list of trial sites based on the date when they joined the ARK network, divided into groups of 6, with the implementation date for each organisation then randomly assigned by the randomisation list (i.e randomisation stratified by these blocks). This is feasible, because all research data on which the intervention is evaluated are routine electronic health record data that are completely independent of the trial. Thus, they are available historically and collected in the same way, regardless of the date of randomisation/implementation— there are no research-specific outcome data in the trial (details in a subsequent section). Organisations have no control over which block they are allocated to or to their randomisation date: 24 organisations had already joined the network at the start of randomisation. This was a pragmatic decision based on the fact that trusts in the main trial would be randomised over 18 months, and in the current NHS climate it is impossible to get NHS trusts to agree to join a programme to reduce antibiotic use where they might not get the intervention for 18 months. All comparisons will be made before and after implementation in the same trust (see the following paragraphs), since any comparison across trusts pooling time will be irretrievably confounded by various organisational changes internally and externally.

In practice, the local champions need some lead time to get an implementation team together, to identify the essential individuals who drive prescribing decisions for acute/general medical inpatients at the organisation (in whom training uptake will be assessed) and to arrange meetings with staff before implementation. Randomisation therefore occurs 3 months before the target ‘implementation date’ (baseline), and the implementation toolkit contains a focused set of activities from this randomisation date through to implementation.

Randomisation uses computer-generated random numbers and is based on two to four organisations implementing per month (on Mondays), avoiding implementation dates over the summer holidays, Christmas and Easter. The allocation sequence has been generated by the Trial Statistician (ASW) and is concealed from everyone except the Trial Statistician. The Trial Statistician informs the Trial Manager of the next randomisation 5 days before the randomisation by email; the Trial Manager then informs the site by email on the randomisation day. Any organisation which withdraws from the trial between randomisation and randomised implementation date will be replaced, and their electronic research data will not be collected. They will be reported as a withdrawal.

### Follow-up

The intervention is delivered to healthcare professionals involved in prescribing or administering antibiotics, but outcomes are assessed in inpatients admitted to acute/general medicine (see the following section). Therefore, follow-up periods relate to calendar time before and after implementation intervention. Routinely collected electronic patient health data are therefore requested from each organisation from 1 February 2016 (2 years before the start of phase III), or as close to this as possible (or up to 2 years pre-implementation for feasibility and pilot organisations). Final data will be requested on 31 October 2020, 15 months after the last randomised implementation date, to allow 90-day mortality to be ascertained on the last patients admitted up to 12 months’ post the last randomised implementation date.

### Outcomes and their assessment

The main primary and secondary outcomes will be assessed using anonymised routine electronic health records data that are already captured at participating organisations. These data will be extracted from NHS databases and anonymised by local organisation informatics/information technology departments (funding is provided to support this) before sending to the research team, following a pre-defined data specification document detailing data items and coding. All patients can request that their NHS records not be released for secondary use (for example, in Hospital Episode Statistics (HES)). Patients who have opted out of sending their hospital records to NHS Digital will not have information submitted for intervention evaluation.

The phase III main trial has two co-primary outcomes:

- 30-day mortality post-admission (non-inferiority)
- DDDs of antibiotics per acute/general medical admission (superiority).

Admission (rather than bed-day) is the denominator for this co-primary outcome (similar to the DH Antimicrobial Prescribing Quality Measures), because bed-day can be strongly influenced by non-medical reasons for not discharging inpatients [46, 47]. The reason for including two co-primary outcomes is to include both a patient health outcome, where we expect to see no evidence of benefit from using fewer antibiotics, but importantly no evidence of harm; and also an endpoint reflecting the overarching goal of the intervention, namely to reduce antibiotic use. However, the full trial will not evaluate a shorter course of antibiotics per se, but an intervention designed to reduce antibiotic duration enacted through healthcare professionals’ behaviour. There is therefore no guarantee that it will have the desired effect on total antibiotic usage: if the intervention does not reduce antibiotic consumption, de facto it will have failed. For this reason, antibiotic consumption is a co-primary superiority endpoint. The fact that the primary endpoint (30-day mortality) is for non-inferiority is another reason for including antibiotic duration as a co-primary endpoint; the intervention will be considered successful only if it significantly reduces antibiotic usage with no evidence of increased mortality.

No personal data are collected, but as date of death is considered confidential (but not personal as it does not relate to a living individual), approval has been obtained from the Research Ethics Committee (REC) and the Confidentiality Advisory Group (CAG) for data collection without individual consent.

The primary and secondary outcomes (Table 1) will be supplemented by additional quantitative, qualitative and laboratory data in order to assess uptake, use and other impacts of the intervention (also listed in Table 1). Gut flora resistance potential will be assessed using repeated point prevalence surveys at three organisations, following the stepped-wedge randomised design using freshly discarded faecal samples remaining after *Clostridium difficile* testing. Diarrhoea is very common in acute/general medical inpatients, with multiple causes, the majority not infectious. All inpatients with diarrhoea will have samples sent for *C. difficile* testing, making this a relatively generalisable reflection of the population, particularly of those patients likely to provide the greatest contribution to spreading resistance elements by the oral-faecal route (because they have diarrhoea) and who have received antibiotics (because antibiotics can cause diarrhoea). Less than 5% test positive for *C. difficile*; that is, the actual disease burden is low. Samples will be identified for the study by staff in the routine service microbiology laboratory. They will be analysed for gut flora ‘resistance potential’ without respect to any patient-identifying information, and ethical approval has been obtained to do this without individual patient consent. No single method is currently available to estimate the ‘resistance potential’ of gut microorganisms. Culture is specific to particular organisms and is practically feasible for only a small number of species, excluding any that are non-culturable by default. Targeted molecular approaches, such as polymerase chain reaction (PCR), can only identify a subset of thousands of known variants of antimicrobial resistance (AMR) genes/mutations in a sample [48]. Because of this, we plan to use metagenomic sequencing directly from extracted faecal DNA to characterise the gut ‘resistance potential’, comparing the millions of short sequences produced to catalogues of thousands of AMR genes, and theoretically providing a quantitative and representative estimate of ‘resistance potential’ [49, 50].

**Table 1:** Primary, secondary and other objectives and measures in the main trial

Objectives	Outcome measures	Timepoint(s) of evaluation of this outcome measure
Co-primary objectives		
To compare the effect of introducing the behavioural intervention on 30-day mortality post-admission as an acute/general medical inpatient (non-inferiority)	Death, ascertained from hospital records which are routinely updated from national death reporting	30 calendar days after admission to acute/general medicine
To compare the effect of introducing the behavioural intervention on antibiotic exposure per acute/general medical admission (superiority)	Defined daily doses (DDDs) of antibiotics per acute/general medical admission, ascertained from electronic hospital prescribing records	Over each acute/general medical admission, including antibiotics prescribed at discharge
Secondary objectives		
To compare the effect of introducing the behavioural intervention on antibiotic exposure using different metrics	DDD per occupied bed-day, days on antibiotics per admission and bed-day (length of therapy (LOT)),^a^ antibiotic days per admission and bed-day (days of therapy, DOT^a^), carbapenem DDD, DOT^a^ and LOT^a^ (per admission and per bed-day), broad-spectrum DDD, DOT^a^ and LOT^a^ (per admission and per bed-day), IV and oral DDD, DOT^a^ and LOT^a^ (per admission and per bed-day), WHO-defined ’Access’, ’Watch’ and ’Reserve’ DDD, DOT^a^ and LOT^a^ (per admission and per bed-day), ’Access’ as a percentage of all antibiotic use	Over each acute/general medical admission, including antibiotics prescribed at discharge
To compare the effect of introducing the behavioural intervention on adverse outcomes	ICU admission during the current admission, total length of stay (hours), antibiotic restart after discontinuation,^a^ emergency re-admission in the 30 days after discharge, *C. difficile* diarrhoea, mortality over the longer term	ICU admission over the current admission; for emergency re-admission, up to 30 days post-discharge; for *C. difficile* diarrhoea, up to 90 days post-admission; for mortality up to 90 days post-admission
To evaluate the impact of the intervention on the faecal flora	Proportion of discarded faecal samples from medical inpatients from which extended spectrum beta-lactamase (ESBL)-carrying *Enterobacteriaceae* can be isolated	Repeated cross-sectional surveys over time
Tertiary objectives		
To evaluate the cost-effectiveness of the behavioural intervention	Resource utilisation and costs	Over the current admission and up to 30 days post-discharge
To quantify uptake and acceptability of the online training component of the behavioural intervention (process outcome)	Proportion of locally identified and pre-specified essential individuals who drive prescribing decisions for acute/general medical inpatients at the organisation who complete the online training (recorded electronically by individuals entering their name and work (NHS) email on the last page) or on an attendance list if the local organisation has opted to do face-to-face training sessions	Within 3 months of invitation to complete training
To quantify uptake of the ARK ‘review and revise’ procedure within the behavioural intervention (process outcome)	Proportion of regularly audited antibiotics prescriptions which document the ARK classification criteria Proportion of regularly audited antibiotics prescriptions which are stopped at ‘review and revise’	Over 12 weeks following implementation date

^a^In organisations with individual electronic prescribing (rather than bulk) antibiotic data

Note: WHO definitions of ’Access’, ’Watch’ and ’Reserve’ antibiotics will be used, following the 2017 Essential Medicines List, using the PHE interpretation. Broad-spectrum is defined as co-amoxiclav; piperacillin/tazobactam; second (e.g. cefuroxime), third (e.g. ceftriaxone ceftazidime) or fourth (e.g. cefepime) generation cephalosporins or cephalosporin-beta-lactamase inhibitor combinations (e.g. ceftazidime-avibactam, ceftolozane-tazobactam); carbapenems; quinolones; azithromycin; tigecycline; aztreonam; and telithromycin. Cefaclor is not included as a second generation cephalosporin because it is administered orally and is not well absorbed

Process evaluation will also include interviews/focus groups with healthcare professionals, a brief questionnaire concerning healthcare professionals’ perceived barriers to review and revise (collected online on completion of the online training and 1 month later) and healthcare professionals’ usage of the online training (collected automatically by the website and mobile software). Implementation of ARK will also be assessed using a questionnaire completed by the ARK champion.

There is intense interest in the use of electronic health records in clinical trials [51]. However, they also have important challenges, three being notable for the ARK intervention: defining the intervention population; assessment of antibiotics; and access.

Firstly, the population receiving the intervention is (adult) acute/general medical inpatients. However, in practice different organisations have different patient pathways; some organisations retain patients in acute medical units for the duration of their admission, whereas in others they swiftly move to related specialties such as gastroenterology, gerontology, respiratory, etc. Part of the implementation toolkit involves mapping these patient flows and involving all relevant clinicians in training. However, such acute/general medical inpatients are not always identified by the use of the 300 (general medicine) specialty code (Fig. 1). In 2017 the Society for Acute Medicine and the Nuffield Trust (Martin Bardsley, personal communication) completed an exercise identifying the most commonly used specialty codes under which adult acute/general medical inpatients are admitted, and we follow their definition. The patient population is therefore defined by either the treatment specialty code (under which the patient is treated) (HES “tretspef”) or the main specialty code (of the consultant) (HES “mainspef”) of either the first or second consultant episode for an inpatient spell, where the inpatient spell has the primary admission date (HES “admidate”) during the time periods specified previously. Paediatric admissions have different specialty codes and are not included in this definition of the analysis population (and are not exposed to the intervention, which is implemented in services admitting adult acute/general medical inpatients).

Because of the potential for exposure misclassification (which may vary by organisation), sensitivity analyses of the primary outcomes will be conducted excluding cardiology (320), rheumatology (410), haematology (303) and neurology (400). If results provide a qualitatively different interpretation, these sensitivity analyses will be conducted for all outcomes.

DDDs using standard WHO formulae (www.whocc.no) are designed to transform milligrams of different antibiotics into standard units reflecting a typical dose and are recommended for use in antibiotic evaluations [52]. Their most important limitation is that reducing prescribing of broad-spectrum antibiotics often involves moving to a combination of multiple agents of narrower spectrum. A single DDD of a broad-spectrum antibiotic may therefore be replaced with multiple DDDs of narrower spectrum antibiotics, but this is probably better for future antimicrobial resistance. For this reason, where organisations are able to provide individual-level antibiotic prescribing data, we will also analyse length of therapy (LOT), defined as the time (days/hours/minutes) between the last and first administration of antibiotic treatment (lumping all antibiotics together), and days of therapy (DOT), defined as the sum of the time (days/hours/minutes) between the last and first administration of each separate antibiotic. However, relatively few organisations currently have individual-level electronic prescribing systems, and therefore most are only able to provide bulk-level DDDs in acute/general medical inpatients.

Admission (rather than bed-day) is the denominator for antibiotic usage (similar to the UK DH Antimicrobial Prescribing Quality Measures), because bed-day can be strongly influenced by non-medical reasons for not discharging inpatients. However, this provides a challenge since it is not straightforward to identify these admissions from the electronic inpatient admission records (see the preceding discussion). Where hospitals provide data on bulk prescriptions only (e.g. using the JAC or Rx-info systems), they are able to provide both total DDDs and DDDs per admission to the relevant hospital areas. The primary analysis will use this denominator wherever available. Where these data are not available, we will use estimates of the relevant admissions and overnight stays from the population defined above.

The last challenge is obtaining the data from individual organisations. Most do not have single unified systems, so patient admissions, antibiotic usage (bulk DDDs or individual level prescriptions) and *C. difficile* data are all held on separate databases and have to be extracted, merged and pseudo-anonymised by overworked information technology departments. This is proving to be a significant challenge despite £14,000 per organisation being provided to obtain these data four times over the course of the trial (for Data Monitoring Committee [DMC] meetings and final analysis).

### Statistical analysis

Analyses will be presented for all organisations. However, the primary analysis will include only the pilot and main trial trusts (the pilot trusts are included to maximise power and because the intervention was essentially unchanged between the pilot study and the main trial, and the pilot study sites did not choose their implementation dates). A sensitivity analysis of the primary outcomes will include the main trial sites only. If results provide a qualitatively different interpretation, these sensitivity analyses will be conducted for all outcomes.

The primary analysis method for binary outcomes will be logistic regression. This is done because, for mortality, data will be considered to be completely ascertained on the basis of routine checks that each organisation conducts against the national death registry, for example, to ensure that follow-up letters are not sent to patients who have died. Therefore, patients not recorded as dying in the national system will be assumed to be alive 30 days (co-primary outcome) and 90 days (secondary outcome) after admission. The death registry is not up to date in real time, and routine checks are only carried out periodically; the final data extract will be requested 6 months after the last included admission to ensure that ascertainment is as robust as possible. In-hospital mortality is completely ascertained by discharge codes. Further, for all binary outcomes, event rates are relatively low (5–10%), so there is little difference between analysing a cause-specific hazard or a competing risks subhazard. Analysing the outcome using logistic regression also facilitates visualisation of the estimated pre- and post-implementation trends, and the step change at implementation, against the observed percentages for each outcome per organisation. Sensitivity analyses will consider these as time-to-event outcomes and will consider competing risks from inpatient and out-of-hospital deaths as relevant for each specific outcome [53–55]. Negative binomial regression will be used to model antibiotic DDDs, incorporating over-dispersion and using different offsets as defined previously. Other antibiotic outcomes are planned to be analysed using ordinal logistic regression; however, if, for example, data are approximately normally distributed, either before or after transformation, linear regression may be used instead. Box-Cox-transformed length of stay will be analysed using normal linear regression. A sensitivity analysis will include stay only up to the point a patient was declared medically fit for discharge. A second sensitivity analysis will use quantile regression of the 90th percentile to assess effects at the extremes of the distribution.

For analyses comparing periods before and after introduction of the intervention, we hypothesise that the intervention could produce either a step change in each outcome, or ongoing changes over time (i.e. an interaction between intervention vs control and calendar time), or both. Analysis will therefore use an interrupted time series (ITS) analysis which not only estimates whether the intervention has a direct immediate impact, but also whether it has any impact on year-on-year trends after implementation (and compared with year-on-year trends pre-implementation) (Fig. 2). For example, even if there is no immediate impact of the intervention on mortality, it will be particularly important to monitor, say, whether any calendar year trends suggest mortality increases post-implementation. It will also be important to monitor whether antibiotic use declined faster post- vs pre-implementation.

Other reasons for using the ITS approach within organisation, and then pooling organisation-level estimates, are that many other things are likely to be changing differently in different organisations, and within organisations, over calendar time. For example, financial incentives could be introduced to reduce antibiotic use in hospital and increase the numbers of antibiotic prescriptions that receive review (under the Commissioning for Quality and Innovation [CQUIN]) payments framework). These external factors will affect within-organisation comparisons less than between-organisation comparisons. Further rates may not be constant before or after the intervention. Finally the calendar-time blocked randomisation makes the assumptions underlying the standard vertical comparisons (between organisations already randomised to intervention vs not at each step) more questionable. The date of introduction of the intervention will be the planned date of implementation for the feasibility and internal pilot organisations and the date that each organisation was randomised to implement the intervention in the main trial. Sensitivity analyses for the main trial will also consider the fact that implementation may occur gradually over approximately 3 months [56], by incorporating a trend for the 3 months post-implementation date instead of a single step change at the implementation date. The DMC will be asked to advise on the most appropriate analysis model as part of their Charter.

Each outcome will first be modelled separately in each organisation, estimating trends before and after intervention implementation, plus an implementation-associated immediate effect (‘step change’) (Fig. 2). The unit of analysis will be admission. Patient will be used as a clustering variable for robust variance adjustment (but not otherwise adjusted for). Based on data available to date, we do not expect strong seasonal effects in the outcomes above, but this will be investigated and included if necessary. Meta-analytic techniques will then be used to combine these organisation-specific estimates to produce an overall intervention effect, assessing heterogeneity using *I*^2^ statistics. This approach is similar to a multilevel model, but it has more flexibility in modelling individual organisations. The primary comparison will be a 2 degree of freedom test, jointly testing that the ‘step change’ is zero and that there is no change in calendar trends post- vs pre-implementation. This joint test will be fitted on model parameters and explicitly account for correlation between the two estimands. Secondary analyses will consider calendar trends post-implementation, and change in calendar trends post- vs pre-implementation, and a trend for implementation over 3 months rather than a ‘step change’.

The major concern about unadjusted analyses is the potential for variation in case mix, particularly over a calendar year, to affect estimates of intervention effect. Additional sensitivity analyses will adjust for case mix to assess whether changes in case mix could confound any changes in antibiotic use observed over time. The following admission-level covariates have been chosen based on their significant effects on 30-day mortality in previous analyses of emergency admissions from routinely collected data [57] and will be adjusted for in each organisation regardless of statistical significance (with non-linear relationships between covariates and outcomes assessed and incorporated using natural cubic splines): sex; age (years) at admission; immunosuppression; Charlson Co-Morbidity Index (defined using secondary diagnosis codes); interaction between age and Charlson [57]; patient classification; admission method, source, specialty, day of the week, time of day and day of year and interaction between admission day of the week and time of day [57]; number of admissions in previous year (excluding as day case), and ever had a complex admission.

All analyses will be modified intention-to-treat, including all randomised organisations who attempted to implement the intervention but excluding any organisations who withdrew between randomisation and randomised implementation date (who therefore never attempted to implement the intervention and in whom effects will be null by definition). It is not possible to specify an individual-level per-protocol population since the precise method of management of each individual inpatient is unknown. All results will be interpreted in the light of the changes in antibiotic use achieved, both overall and by organisation. At the organisation level, a per-protocol population will be defined broadly by > 50% of locally identified and pre-specified essential individuals who drive prescribing decisions for acute/general medical inpatients having accessed the online training (recorded electronically by individuals entering their name and work (NHS) email on the last page or on an attendance list if the local organisation has opted to do face-to-face training sessions). More specifically, we specify eight criteria defining implementation fidelity:

1. Provision of a list of key essential people, by implementation date
2. Achieving at least 20 people per 100 acute beds who have done the online learning, by end of implementation date
3. Introduction of the ARK categories into the prescribing process, generally by adjusting the physical drug chart or e-prescribing system. As a minimum, documentation of categories into clerking or system to force stop/re-prescribe by 72 h in e-prescribing, by implementation date
4. Process in place for making patient leaflet available to acute medical patients, by implementation date
5. Submission of baseline audit data, by implementation date
6. Process in place for ongoing audit and feedback, by implementation date
7. Submission of initial post-implementation audit data, by week 4
8. Submission of electronic patient research data, by week 16 following implementation

Failing to meet five or more criteria would be considered a failure to implement in analysis.

Intervention uptake will be assessed by estimating the proportion of locally identified and pre-specified essential individuals who drive prescribing decisions for acute/general medical inpatients at the organisation who complete the online training.

Process evaluation analyses will seek to better understand how ARK was implemented across different hospitals, potential barriers and facilitators and also how implementation influenced use of the intervention and antibiotic use. This will be triangulated with data from qualitative interviews/focus groups, which will undergo thematic analysis. Secondary analyses will consider how much of any effect of the intervention could be mediated through different aspects of the implementation of review and revise, including training completion rates, evidence of implementation from audit records as well as success of implementation in each hospital, based on mixed methods evaluation and documentary evidence, and number of the preceding criteria met.

For the primary endpoint, subgroup analyses will be conducted by investigating heterogeneity in the organisation-level intervention effect estimates across the following factors, using meta-regression techniques: randomisation block (categorical stratification factor), calendar period of randomised implementation date (reflecting different NHS pressures) (Jan–Mar, Apr–Jun, Jul–Sep, Oct–Dec), organisation type (small, medium, large, teaching), region, functional role of champion (acute medicine, microbiology/infectious diseases, pharmacist), paper vs electronic prescribing systems, percentage of essential people completing training by 12 weeks, total number completing training by 12 weeks per 100 acute beds.

### Missing data

Other than antibiotics, outcome data and variables for adjustment are based on routine electronic health records linked by NHS numbers within participating organisations. As these records are used for patient management, data in the variables being requested should not be missing. As above, for mortality, data will be considered to be completely ascertained; similarly, for other outcomes, as follow-up time is relatively short, observed data will be assumed to represent all events within the prescribed follow-up time (30–90 days).

For adjustment variables, first if demographics are missing for only some records for a patient, these will be carried forwards and backwards to any other records with missing values. Afterwards, any records with missing values for any of the key adjustment variables will be enumerated. If these comprise < 0.1% of records from an organisation, they will be assumed to represent incorrect records in the underlying data sources and checked with the individual organisation, but will be dropped from all analyses (adjusted and unadjusted). Where records with missing data comprise > 0.1% of records from an organisation, this will be queried, since it is not anticipated that this should ever occur with the data items above.

### Health economics

A within-trial cost-effectiveness analysis will estimate and compare the costs and impact of ‘review and revise’ compared to standard care. The primary analysis will take an NHS perspective based on data collected within the phase III main trial and supplemented with data from the literature where necessary (e.g. quality-of-life estimates). Costs of inpatient stays will be obtained from the Reference Costs Database (DH). Intervention costs will be estimated from admission and antibiotic data collected in the main trial. Costs of antibiotics used will be estimated from the British National Formulary Drugs and pharmaceutical electronic market information tool (eMIT) for generic antibiotics, and actual costs (negotiated locally) obtained from participating organisations (purposively sampled based on size and type). Collecting such detailed information on actual antibiotic costs is important, because whilst we expect that the intervention would lead to lower antibiotic use, and hence reduced costs, this is not necessarily the case. It might lead to more confidence in prescribing antibiotics initially (e.g. following ‘Surviving Sepsis’ [26]) with appropriate discontinuation at early review. Costs of tests (e.g. C-reactive protein) and investigations (e.g. magnetic resonance imaging (MRI), ultrasound) will be estimated in these same organisations.

Providing the main trial demonstrates the intervention is non-inferior for 30-day mortality and superior for antibiotic usage, we will compare the mean per-patient costs, pre- and post-intervention implementation, and calculate the difference, and then estimate the incremental cost per percentage reduction in antibiotic usage (overall and by organisation). We will then explore alternative thresholds to examine the maximum willingness to pay for a reduction in hospital stays, admissions (and emergency re-admissions), infection recurrences and quality-adjusted life years gained. A strategy will be deemed cost-effective if the incremental cost-effectiveness ratio is under this threshold. Probabilistic sensitivity analysis will address the joint contribution of parameter uncertainty on decision uncertainty and provide adequate estimates of expected cost and effects. This primary analysis will take an NHS perspective based on resource use within secondary care. Differences in resource use outside of hospital are implicitly assumed to be equal before and after the intervention, and therefore excluded from modelling.

### Other analyses

Analysis described above will be conducted on routine electronic health record data submitted from participating organisations. However, for English trusts, data on both antibiotic prescribing and inpatient admissions (not *C. difficile*) is available through the English Surveillance Programme for Antimicrobial Utilisation and Resistance (ESPAUR), through purchasing data provided to Public Health England (PHE) from a commercial company and through HES respectively. This would enable us to conduct analyses of mortality, re-admission, length of stay and DDDs for all trusts in England, according to whether or not they had implemented ARK within the trial, to compare pre-implementation trends in all English trusts, and step change and post-implementation trends in ARK hospitals vs non-ARK hospitals for these outcomes.

### Sample size

For the phase III main trial, based on simulation studies, the proposed stepped-wedge cluster-randomised design including data from a minimum of 36 organisations has > 85% power to exclude a 5% relative increase in 30-day mortality and to detect a 15% relative reduction in antibiotic use associated with the intervention. The 5% relative non-inferiority margin means that to declare non-inferiority, the entire 95% confidence interval (CI) for the relative change in mortality post- vs pre-intervention has to lie within [0.95,1.05]. For an overall mortality of 9%, this is analogous to being confident that the post-intervention mortality lies within [8.5%, 9.5%], that is, a non-inferiority margin of +/− 0.5% on the absolute scale or 5% on the relative risk or relative odds scale (which are similar given the low event rates).

This sample size calculation was based on simulation, given the uncertainties regarding parameters underlying the study design. Simulations were run 1000 times for *N* = 10 to 40 participating organisations in increments of 2 organisations and a relative decrease in antibiotic use of *R* = 5% to 20% in increments of 5%. Organisations were assumed to vary in size with an underlying rate of acute/general medicine admissions per year from 5000 to 15,000 (uniform distribution), plus one large organisation of 25,000 admissions per year (estimates based on acute/general medical admissions in Oxford University Hospitals (OUH) NHS Foundation Trust, and publicly available data on the distribution of annual overnight stays in all English NHS trusts). Before the intervention is introduced, each organisation is assumed to have an underlying rate of 1000 DDDs per acute/general medical admission drawn from a uniform distribution on 1500–3000. Each organisation’s 30-day mortality is assumed to be distributed uniformly between 7 and 10%, independently of antibiotic prescribing. The intervention was assumed to decrease DDDs per 1000 admissions by *R*% with no effect on mortality: there are no time trends before or after implementation in either outcome. The intervention is implemented in two randomly chosen organisations every month for 18 months, followed by a further 12 months post-intervention follow-up. Each month, the number of acute/general medical admissions is simulated using a Poisson distribution based on the underlying rate simulated as above. Each month, DDDs are then simulated from a negative binomial distribution based on the simulated number of admissions that month, each organisation’s simulated underlying prescribing rate and whether the month falls before or after the randomly allocated intervention time. The dispersion parameter is fixed at 1.15 times the mean, slightly higher than the estimated dispersion from monthly antibiotic prescribing in OUH acute/general medical inpatients [33]. Each month, the 30-day mortality is then simulated from a binomial distribution based on the simulated number of admissions that month, with a probability drawn from a normal distribution centred on each organisation’s simulated underlying mortality rate with standard deviation 0.1. Analysis follows the above-described methods (and Fig. 2). No sample size inflation was applied to the two co-primary outcomes because the goal of the non-inferiority comparison is to demonstrate that the 95% CI around the estimated relative risk is within a pre-defined non-inferiority margin, rather than to identify a significant difference.

### Ethical issues

As described above, patients who have opted out of sending their hospital records to NHS Digital would not have information submitted for intervention evaluation. A generic poster describing the study is provided to participating organisations to be displayed in emergency departments, clinical decision units, acute medical units, etc., where included patients will be managed. The poster contains a link to the study’s public webpage, where more information about the study is provided in lay language, and a study-specific email address, through which patients can contact the research team. The poster also contains details of the local Principal Investigator or another local NHS professional whom patients can contact if they have questions or wish to opt out of data provision. Removing patients’ data would require them to provide identifiable data on their NHS number and hospital number. The research team do not hold any identifiable data; therefore, they would direct such a patient to their organisation’s local Principal Investigator to ensure the patient’s data are removed at source at the participating organisation before anonymised extracts are sent to the central research team.

### Oversight

An independent DMC will review outcome data from the phase III main trial approximately 6 months after it starts and then approximately every 9 months. The DMC is advisory: after each meeting they will make a recommendation that the trial continue, or be stopped or modified to the Programme Steering Committee which will meet every year throughout the programme. The DMC will consider both clinical and statistical evidence. Statistical evidence would be based on the Haybittle-Peto rule (*p* < 0.001 for impact of the intervention): this has the advantage that the number of DMC meetings does not have to be pre-specified in advance, and the DMC can meet more frequently as determined by the data. The DMC would consider both co-primary outcome measures in their deliberations regarding trial stopping. Further details of DMC functioning and the procedures for interim analysis and monitoring will be specified in a DMC Charter. The Programme Steering Committee is the executive body. This independent oversight committee is termed a Programme Steering Committee rather than a Trial Steering Committee because the trial is part of a larger programme, and the Programme Steering Committee provides consistent independent oversight across the entire programme of related work.

### Main trial status

The first organisation was randomised in the main trial on 13 November 2017 (under protocol version 4.0) to implement on 12 February 2018. The last organisation is planned to be randomised on 1 April 2019 to implement on 24 June 2019.

The current protocol is version 5.0 (5 November 2018). All protocols have been approved by the South Central Oxford C Research Ethics Committee (REC) (17/SC/0034) and the Confidentiality Advisory Group (CAG) (17/CAG/0015). Protocol 1.0 (16 December 2016) was submitted for REC and CAG approval for the three phases of the study. Version 2.0 (24 March 2017; phase I feasibility study) incorporated clarifications requested by REC and CAG including those on opt-out and the electronic health records analysis population, and increased preparation time from randomisation to implementation from 3 to 4 months based on experience preparing for the feasibility study. Version 3.0 (25 September 2017; phase II pilot study) included further clarifications on the analysis population based on advice from the Nuffield Trust and extension of process evaluation to include interviews. Version 4.0 (6 November 2017; phase III main trial) returned preparation time from 4 to 3 months based on experience in the internal pilot and removed an acceptability rating which had not been collected. Version 5.0 (5 November 2018) increased the number of organisations included in process evaluation and clarified how individuals would be recruited for these qualitative studies, adding eight criteria defining implementation fidelity and several other clarifications regarding objectives, outcome measures, withdrawal, population and analysis to align with the statistical analysis plan version 1.0.

### Reporting

Final results will be reported according to the Consolidated Standards of Reporting Trials (CONSORT) extension for stepped-wedge cluster-randomised controlled trials [58].

### Data sharing and dissemination

The full protocol is available on http://www.arkstudy.ox.ac.uk/ark-for-healthcare-professionals/. Over the 4.5 years of the study, we anticipate including more than 1 million inpatient admissions from 36 organisations. Given the size of the final dataset, we have therefore not requested or obtained permission from all the involved organisations for it to be shared outside the trial team. Dissemination will include publication in a peer-reviewed journal and presentation at conferences. Authorship will be determined in accordance with the International Committee of Medical Journal Editors (ICMJE) guidelines, and other contributors will be acknowledged. The ARK intervention has been endorsed by key societies including the British Society for Antimicrobial Chemotherapy (BSAC), the Society for Acute Medicine, the UK Clinical Pharmacy Association and the British Infection Association. In collaboration with Health Education England, BSAC have worked with the trial team to develop a post-trial version of the intervention which is freely available to all NHS Trusts and Health Boards (https://portal.elfh.org.uk/Component/Details/582663 and http://bsac-vle.com/ark-the-antibiotic-review-kit/).

## Additional file

Additional file 1: SPIRIT 2013 checklist: recommended items to address in a clinical trial protocol and related documents. (DOC 160 kb)

## Data Availability

Not applicable.

## Abbreviations

ARK: Antibiotic Review Kit
BSAC: British Society for Antimicrobial Chemotherapy
CAG: Confidentiality Advisory Group
CI: Confidence interval
DDD: Defined daily dose
DMC: Data Monitoring Committee
DOT: Days of therapy
ESBL: Extended spectrum beta-lactamase
ESPAUR: English Surveillance Programme for Antimicrobial Utilisation and Resistance
HES: Hospital Episode Statistics
ITS: Interrupted time series
LOT: Length of therapy
NHS: National Health Service
NIHR: National Institute for Health Research
OUH: Oxford University Hospitals
PHE: Public Health England
REC: Research Ethics Committee
